# Dual Roles of Coconut Oil and Its Major Component Lauric Acid on Redox Nexus: Focus on Cytoprotection and Cancer Cell Death

**DOI:** 10.3389/fnins.2022.833630

**Published:** 2022-03-11

**Authors:** Venkatesan Ramya, Karuppiah Prakash Shyam, Eshwaran Kowsalya, Chelladurai Karthikeyan Balavigneswaran, Balamuthu Kadalmani

**Affiliations:** ^1^Reproductive Endocrinology and Cancer Biology Laboratory, Department of Animal Science, Bharathidasan University, Tiruchirappalli, India; ^2^Research and Development Division, V.V.D and Sons Private Limited, Thoothukudi, India; ^3^Tissue Engineering and Biomaterials Laboratory, Department of Biotechnology, Bhupat and Jyoti Mehta School of Biosciences, Indian Institute of Technology Madras, Chennai, India

**Keywords:** neuroinflammation, extraction process, lauric acid (LA), antioxidants, reactive oxygen species, cellular redox homeostasis, coconut oil (CO)

## Abstract

It has been reported that coconut oil supplementation can reduce neuroinflammation. However, coconut oils are available as virgin coconut oil (VCO), crude coconut oil (ECO), and refined coconut oil (RCO). The impact of coconut oil extraction process (and its major fatty acid component lauric acid) at cellular antioxidant level, redox homeostasis and inflammation in neural cells is hitherto unexplained. Herein, we have shown the antioxidant levels and cellular effect of coconut oil extracted by various processes in human neuroblastoma cells (SH-SY5Y) cultured *in vitro*. Results indicate VCO and ECO treated cells displayed better mitochondrial health when compared to RCO. Similar trend was observed for the release of reactive oxygen species (ROS), key oxidative stress response genes (GCLC, HO-1, and Nqo1) and inflammatory genes (IL6, TNFα, and iNOS) in SH-SY5Y cells. Our results signified that both VCO and ECO offer better neural health primarily by maintaining the cellular redox balance. Further, RCO prepared by solvent extraction and chemical refining process lacks appreciable beneficial effect. Then, we extended our study to find out the reasons behind maintaining the cellular redox balance in neuroblastoma cells by VCO and ECO. Our GC-MS results showed that lauric acid (C14:0) (LA) content was the major difference in the fatty acid composition extracted by various processes. Therefore, we evaluated the efficacy of LA in SH-SY5Y cells. The LA showed dose-dependent effect. At IC_50_ concentration (11.8 μM), LA down regulated the oxidative stress response genes and inflammatory genes. The results clearly indicate that the LA inhibited the neuroinflammation and provided an efficient cellular antioxidant activity, which protects the cells. The efficiency was also evaluated in normal cell line such as fibroblasts (L929) to cross-validate that the results were not false positive. Different concentration of LA on L929 cells showed high compatibility. From our observation, we conclude that VCO and ECO offers better cellular protection owing to their powerful antioxidant system. Therefore, we advocate the inclusion of either VCO and/or ECO in the diet for a healthy lifestyle.

## Introduction

Dietary fats are one of the main sources of energy for cells. Recently, remarkable interest was generated in the effect of dietary fats on health benefits of neural disorders (Chianese et al., 2018), cardiometabolic diseases (Kadalmani et al., 2011; Wu et al., 2019), and type 2 diabetes mellitus (Risérus et al., 2009; Shyam and Kadalmani, 2014). Decades of research indicate complex health effects of individual dietary fats. Earlier we had shown the effect of acarbose and voglibose on serum lipid when combined with sulfonylureas or biguanides in clinical patients with type II diabetes mellitus (Revathi et al., 2011). Interestingly lipids and fat metabolites are involved in various processes including neural development and inflammatory reactions (Das, 2006). Fatty acids are the most important chemical element of fats. Fatty acids (FA) have four major physiological roles. They (i) form building blocks of phospholipids and glycolipids; (ii) acts as an address tag for protein attachment in cell compartment, (covalent attachment of fatty acids to protein targets them to membrane location); (iii) serves as hormones and intracellular messengers; (iv) fuel molecules for the energy, which are stored as triacylglycerols (generally referred to as triglycerides which are uncharged esters of fatty acids with glycerol) (Veeramani et al., 2014). Triglycerides are classified into two types based on the number of fatty acids such as medium-chain triglycerides (MCT’s) (FA with 8–12 carbons) and large-chain triglycerides (LCTs) (>12 carbon FA). The MCTs are absorbed *via* both the portal and lymphatic routes. Because of the low chain-length, the MCTs do not require any additional modifications or bile salt digestion for absorption. They passively diffuse from the GI tract to the portal system. However, as chain length increases from 12 to 14 carbons for LCT, the major route of transport occurs through the lymph. Thus 12 to 14 carbon chain length is considered to be the point of transition (Maggio and Koopmans, 1982). Patients who have malnutrition, malabsorption, or fatty acid metabolism disorders are treated with MCTs because MCTs do not require energy for absorption, use, or storage. MCT is used by the body differently from the LCT (Pizzorno and Murray, 2020). Unlike LCT, MCT is highly ketogenic and energetically less dense, which are readily oxidized for energy (Krotkiewski, 2001; Auestad, 2008). MCT helps in regulating energy balance and aid in brain development in infants therefore preterm infant formulas usually contain 60% MCTs of the total fat (Auestad, 2008; Wallis et al., 2021). Further, in aged dogs dietary supplementation with MCT had been shown to improved cognitive function (Pan et al., 2010). Long-term MCT increases the circulating ketone levels. Circulating ketones are considered as an alternative energy source for the brain (Pan et al., 2010).

Coconut oil is the richest natural source of MCT that could contribute to the health benefits by increasing metabolism rapidly due to their shorter carbon chains (Carandang, 2008; Dayrit, 2014). In addition, coconut oil is reported to have additional health benefits owing to its properties such as anti-infective (Strunk et al., 2018), antimicrobial (Joshi et al., 2020), and antiviral effect (Ogedengbe et al., 2018). Coconut oils are available as virgin coconut oil (VCO), crude coconut oil (ECO, prepared by expeller process), or refined coconut oil (RCO, prepared by solvent extraction and chemical refining process) (Chakrabarty, 2003). Of note solvent extraction process is also used in the essential oil extraction procedures (Venkatesan et al., 2019). The differences between these oils are their extraction processes and subsequent changes in their fatty acid levels (Liu et al., 2019). VCO is a new high-value-added version of coconut oil (Naik et al., 2015). Despite the high cost, VCO is considered and marketed as one of the “healthiest oil.” VCO contains 48–53% of lauric acid (LA). Interestingly, lauric acid is reported to improve digestion and metabolism, help to stimulate immunity and contribute in balancing better serum lipid profile (Dumancas et al., 2016; Narayanankutty et al., 2018). Although the health benefits of VCO are reported, the impact of the extraction procedure was not explored well. Furthermore, threshold limit of the presence of LA, which is major constituting element in VCO has not been reported so far. Herein, we had studied the impact of different oil extraction processes on the cellular redox state. We had used SH-SY5Y cells to study the impact of oil obtained *via* different extraction processes and studied the effect of LA in altering the cellular redox balance. SH-SY5Y is a human-derived cell line that is widely used as an *in vitro* model to study neuronal function, differentiation, neurodegenerative disorders such as Parkinson’s disease (PD), Alzheimer’s disease (AD), neurogenesis, and other characteristics of brain cells. In neurological research, differentiated and un-differentiated SH-SY5Y cells are frequently used because they respect their neuronal and dopaminergic characteristics, especially in PD (Ahmi, 2019). Most importantly, differentiated SH-SY5Y cells show tyrosine hydroxylase and dopamine-β-hydroxylase activities that are critical in PD research. In addition, several studies had proven that oxidative stress and mitochondrial dysfunction are significant factors in the pathogenesis and development of PD (Salari and Bagheri, 2019), these effects can be well studied using SH-SY5Y cells *in vitro*. Of note, the cell line was originally derived from a bone marrow biopsy of a 4-year old female neuroblastoma patient.

Here, we approach and interpret the cellular behavior on two perspectives. (a) Understanding the molecular players that facilitate growth and progression of the neurons because PD-related problems manifest abnormal mitochondrial function, oxidative stress, and autophagy, thus this perspective relates cellular oxidative stress, redox balance and neuroinflammation. (b) Understanding the beneficial effects of LA in triggering apoptosis mediated cell death in the cancer microenvironment (exploiting the cancerous nature of SH-SY5Y). Briefly, SH-SY5Y cells were treated with coconut oil (CO) obtained by various extraction processes *viz* ECO, VCO, and RCO. The cell proliferation, differentiation, regulation of key oxidative response genes were studied to identify which oil provides better cellular redox balance in developing neuronal cells. In addition, cells were treated with pure LA at different doses to understand the potential role of LA in altering the cellular redox balance, inflammation and its effect in cancer.

To investigate how CO and LA differentially affect neuroinflammation, we studied the expression of key cytokines such as interleukin-6 (IL-6), tumor necrosis factor α (TNFα), and inducible nitric oxide synthases (iNOS). Cytokines are majorly produced in immune cells such as lymphocytes (B and T cells), monocytes, macrophages, and vascular endothelial cells. However, the key role of cytokines in normal central nervous system function had led researchers to explore the production of cytokines in neural cells and brain, eventually identifying several sources including microglia, pericytes, choroid plexus, astrocytes, microvessel endothelial cells and invading inflammatory cells (Galic et al., 2012). IL-6 is one of the major cytokine expressed by astrocytes, microglia, and neurons and is implicated to play a major role in neurodegeneration (Godbout and Johnson, 2004). Elevation of TNFα is a hallmark of acute and chronic neuroinflammation (McCoy and Tansey, 2008). Similarly, nitric oxide synthases (NOS) is involved in many pathological processes. Therefore, we chose these three major cytokines to assess the inflammatory response. Of note, three NOS isoforms were identified thus far *viz* neuronal NOS (nNOS or NOS1), an inducible NOS (iNOS or NOS2), and endothelial NOS (eNOS or NOS3). Of which, both nNOS and iNOS were soluble NOS, whereas eNOS is a membrane bound NOS. Among the three, both nNOS and eNOS are calcium sensitive while iNOS is calcium-insensitive because of it tight non-covalent interaction with calmodulin (CaM) and Ca^2+^. In addition, NF-κB-dependent activation of the iNOS promoter indicates a possible stimulation of the gene transcript in inflammation condition. Also, iNOS is not normally present in the brain but it can be detected in the brain after inflammatory, infectious or ischemic damage (Sonar and Lal, 2019), thus their expression indicates pathological manifestation in neural cells. Thence, we chose iNOS over nNOS and eNOS in the present study.

## Materials and Methods

### Chemicals, Reagents and Cell Culture

Virgin coconut oil (VCO), crude coconut oil from expeller (ECO), and refined coconut oil (RCO) were kindly provided by V.V.D and Sons Private Limited, Thoothukudi, Tamilnadu. Of note, the ECO oil samples were obtained directly after the expeller process followed by filtration, hence we were able to confirm that ECO were not subjected for any additional process. RCO was procured externally from coconut oil refining plant. All the chemicals used in this study were procured from Merck (India). Eurofins (India) synthesized the gene primers for polymerase chain reaction (PCR) amplification. Hoechst 33258, JC1 fluorescent probe, H_2_DCF-DA, and propidium iodide (PI) were purchased from Thermo Fisher, United States.

SH-SY5Y (ATCC CRL-2266), L929 (ATCC CCL-1) and 3T3-L1 (ATCC CL-173™) cells were obtained from National Centre for Cell Sciences (NCCS), Pune, India and cultured in Dulbecco’s Modified Eagle Medium (DMEM) (Himedia, Mumbai, India) containing 4.5 gm glucose per liter, 25 mM HEPES buffer, L-glutamine, sodium pyruvate and sodium bicarbonate, supplemented with 10% fetal bovine serum (Gibco, United States) and 1% antibiotic solution. The 100X antibiotic solution consists of 10,000 U penicillin and 5 mg streptomycin per ml in 0.9% normal saline (Himedia, India). Cultures were maintained at 37°C, 5% CO_2_ under humid atmosphere in a CO_2_ incubator that has O_2_ controller provision (CellXpert C170i, Eppendorf, Germany). The passage numbers of the SH-SY5Y, L929 and 3T3 cells were 21, 17, and 9, respectively. Upon attaining 80% confluence, the cells were split at 1:3 ratio.

### Physico-Chemical Parameters of Virgin Coconut Oil, Crude Coconut Oil, and Refined Coconut Oil

#### Physical Characterization of Oil

The color of VCO, ECO, and RCO were determined as per the Agricultural produce (Grading and Marking) Act, 1937 (Act. No. 1 of 1937) r/w General Grading and Marking Rules, 1988 Schedule IV Agmark grade designation and definition of Coconut oil (Agmark-Grade-Specifications, 1937; Gupta et al., 2010). The color of the VCO, ECO, and RCO were measured on Lovibond scale using 1-inch cell and expressed as Y + 5R. In general the color for coconut oil (Grade I) should not be deeper than four on Lovibond Tintometer scale. It is to be noted that the color on Lovibond scale should be measured using 1-inch cell for coconut oil. If the color measured is using cells of different size then the results obtained will be erroneous. The Refractive Index of VCO, ECO, and RCO were determined as per the method outlined by AOAC 921.08 (Subroto et al., 2017). Densities of oil was measured using weighing balance by measuring mass and volume in a pre-weighed measuring cylinder (ρ = m/V). The moisture and volatile matter percentage of the oils were determined according to ISO 662:2016 method.

#### Determination of Free Fatty Acid Percentage

The free fatty acids (FFA) content of VCO, ECO and RCO was determined as per the method of ISO 660 or AOAC 940.28. Briefly, neutral methanol was prepared by adding 0.1 N NaOH (pH = 7.0). Oil (7.05 g) was added to the neutral methanol and titrated against 0.25 N NaOH. The titter value was directly proportional to the FFA% (expressed as% equivalent oleic acid).

#### Determination of Iodine Value

We determined the iodine value (IV) according to the procedure outlined by Wijs–ISO 3961. VCO, ECO and RCO (10 g v/v) was added to iodobromine (IBr) in glacial acetic acid (in excess). Potassium iodide (KI) was used to convert the un-reacted IBr to Iodine. The mixture was titrated against standard sodium thiosulphate and the iodine value was determined according Equation 1 presented below.


(1) IV=(b-v)×N×molecularweightofIodine×100/w×1000

where “*b*” is the amount of blank sodium thiosulphate, “*v*” is the amount of sample thiosulphate, “*N*” is the normality of thiosulphate solution, and “w” is the weight of the oil sample (10 g) (Wijs, 1929).

#### Determination of Peroxide Value

To determine the peroxide value (PV), 10 g of oil (VCO/ECO/RCO) were dissolved in acetic acid, followed by chloroform. To the sample, saturated KI mixture was added and the amount of iodine released from the mixture was determined by performing titration against standard sodium thiosulphate. Starch was used as an indicator. The PV was calculated using Equation 2 provided below.


(2) PeroxideValue(PV)=(S-B)×W×N

where “B” is the volume of blank sodium thiosulphate, W is the weight of sample (10 g), “S” is the volume of test (oil) sodium thiosulphate and “N” is the normality of standard sodium thiosulphate (Barthel and Grosch, 1974).

#### Determination of Saponification Value

The saponification value was determined by titrimetry of VCO, ECO, and RCO against 0.5 M hydrochloric acid (HCl) using phenolphthalein as indicator. Prior to titration experiment, the samples were neutralized using KOH. Briefly, 1.0 g of respective oil samples were mixed with KOH (15 ml, 1 N). Subsequently dH_2_O (10 ml v/v, 18 MΩ resistivity) was added to the mixture and refluxed for 30–40 min using Leibig condenser to dissolve the sample completely.

### Chemical Fingerprinting and Fatty Acid Analysis Using Gas Chromatography Coupled With Mass Spectrometry

The VCO, ECO, and RCO samples were pre-processed as described hereunder. 20 mg of VCO/ECO/RCO sample was mixed with 1 ml of methanolic NaOH. The samples were heated at 65°C for 15 min with intermediate shaking. After 15 min, the samples were cooled down to room temperature for 5 min. 1 ml of MilliQ water was added to the mixture followed by addition of 1 ml distilled hexane. The mixture was vortexed and allowed for phase separation. The clear upper layer was gently withdrawn using a micropipette without disturbing the bottom layer. The hexane layer were transferred to a fresh GC vial and subjected to gas chromatography coupled with mass spectrometry (GC-MS) analysis (Ramya et al., 2022). The fatty acids were separated in a HP-88 column using Agilent 7890B gas chromatography operating under following conditions. The samples were injected at injection port held at 250°C. The samples were then transferred to the column through a split injection having 50:1 split ratio. The initial oven temperature was set at 50°C followed by ramping up the temperature to 120°C and hold for 5 min. Further, the temperature was ramped to 170°C at 5°C/min ramp rate and hold for 5 min. Again, the temperature of the oven was ramped to 230°C at 5°C/min ramp rate and hold for 5 min. Finally, the temperature was raised to 250°C at 5°C/min ramp rate and the samples were hold for 2 min. The compounds separated were injected into the Agilent 5899B MSD detector and the total ion chromatogram was recorded in scan mode. The ions ranging from m/z 50 to 500 were recorded (Rajkumar et al., 2018; Shyam et al., 2021a).

### Determination of Antioxidant Activity

The total antioxidant capacity (TAC) was determined as described by Shyam et al. (2021b) with minor modification. The test samples (VCO, ECO, and RCO) were prepared by dissolving 10 mg of oil in 1 ml of ethanol (Shyam et al., 2021b). Hydrogen peroxide scavenging activity of VCO, ECO, and RCO were analyzed as per the method reported previously. Ascorbic acid was used as the reference H_2_O_2_ scavenger (Shyam et al., 2021b). The DPPH radical scavenging activity of VCO, ECO, and RCO were measured spectrophotometrically by 1, 1-diphenyl-2-picrylhydrazyl (DPPH) assay described earlier (Ramya et al., 2021). Similarly, the ABTS• + cation radical scavenging activity of VCO, ECO, and RCO was determined as previously described (Ramya et al., 2021). Experiments were performed in triplicate and the Mean ± S.D were calculated from the results. The half -maximal effective concentration (EC_50_) values were calculated from the observed values using non-linear regression model.

### Cell Based Assays

#### Determination of IC50 by MTT Assay

The IC_50_ concentration of the CO and LA were determined by MTT [3-(4, 5-dimethylthiazol2-yl)-2, 5-diphenyltetrazolium bromide] assay as described earlier (Ramya et al., 2021). Briefly, CO was dissolved in buffer using ethanol as co-solvent. Stock solutions of the test samples (VCO, ECO, and RCO) were prepared by dissolving 1 mg of CO in 1ml of ethanol. Further, test samples were diluted to required concentrations using DMEM media. For LA studies, LA (Sigma Aldrich, United States) was dissolved in ethanol to yield 1mg/ml stock solution. SH-SY5Y cells were seeded in a 96 well plate using DMEM culture medium supplemented with LA at different doses ranging from 0.1 nM to 1 mM for 24, 48, and 72 h. Vehicle (ethanol) served as negative control. Cells without any treatment served as blank (sham). After incubation (at respective time interval) 20 μL of MTT solution (1 mg/mL MTT dissolved in phosphate-buffered saline (PBS)] was added and the plate was incubated for 4 h at 37°C. After 4 h of incubation, the purple color formazan crystals were dissolved using 100 μL DMSO (100%). The absorbance was recorded at 570 nm (sample) and 630 nm (reference) using BioTek Synergy H1MFG microplate reader (BioTek, Agilent Technologies, United States). The experiments were performed in triplicates and repeated three times. The results were presented as means ± SD. The percentage inhibition of the cells was calculated using Equation 3:


(3) $$\begin{array}{c} Inhibition\%=[MeanODofuntreatedcells\left(control\right)\\ -MeanODoftreatedcells]\\ /MeanODofuntreatedcells\left(control\right) \end{array}$$

#### Qualitative Assessment of Cell Viability

Acridine orange (AO) and Ethidium bromide (EB) dual fluorescent staining assay was performed to assess the cell viability and DNA damage (Revathi et al., 2010). Briefly, SH-SY5Y cells were seeded in a six well plate. After attaining 80% confluence, the cells were treated with VCO, ECO and RCO at 0.1 mg/ml (0.2 mM) concentration. The concentration was chosen based upon the circulating free fatty acid (FFA) level in blood. The circulating FFA level ranges between 0.2 to 0.7 mM (Püttmann et al., 1993; Zhao et al., 2016). The cells were incubated for 24 h in the CO_2_ incubator maintained at 37°C and 5% CO_2_. For assessment of cell viability, VCO, ECO, and RCO cells were trypsinized and resuspended in 100 μL PBS. From this, 10 μl of cell suspension was placed onto a microscopic slide, covered with a glass coverslip, and examined. In LA studies, post-incubation (24 h), 1 μl of AO/EB solution was mixed with 25 μl of cell suspension (0.5 to 2.0 × 10^6^ cells/ml) and gently mixed. Each sample were mixed just prior to quantification and evaluated immediately. Cells were viewed under a Leica DMi8 inverted fluorescence microscope (Leica Microsystems, Germany) and counted to quantify apoptosis. DMSO 0.02% was used as a negative control. Apoptosis or necrosis was determined based upon the cytoplasmic organization and nuclear morphology (Gowdhami et al., 2021a,b; Ramya et al., 2021).

#### Assessment of Nuclear Morphology by Fluorescent Nuclear Staining

Nuclear morphology was assessed using Hoechst 33258. Briefly, SH-SY5Y cells were treated with VCO, ECO, and RCO at IC_50_ concentration for 24 h. The control and treated cells were stained using Hoechst 33258 (1 mg/mL). After approximately 3 to 5 min, the cells were suspended onto a glass slide with a cover slip over laid. In experiment involving LA, the adherent cells in the six well plates were imaged directly after washing the stain using PBS. Approximately, 100 cells were evaluated in a Leica DMi8 inverted fluorescence microscope (Leica Microsystems, Germany). The normal and abnormal nuclear morphologies were observed and recorded. Experiments were performed in triplicate (Gowdhami et al., 2021a).

#### Determination of Mitochondrial Membrane Potential (ΔΨm)

The mitochondrial membrane potential (ΔΨm) depolarization was assessed using JC1 fluorescent probe. SH-SY5Y cells were incubated with VCO, ECO, and RCO at the IC_50_ concentration for the respective time interval mentioned thereto. Post treatment, the cells incubated with JC1 mitochondrial membrane potential sensor (7.7 μM) for 20 min. Polarized mitochondria displays red fluorescence. On depolarization, the orange-red punctuate staining is replaced by diffuse green monomer fluorescence. The fluorescence excitation/emission maxima for monomer form and J-aggregate form were 514/529 and 585/590 nm respectively. The fluorescent images were captured using Leica DMi8 inverted fluorescence microscope (Leica Microsystems, Germany) (Ramya et al., 2021). For fluorescence quantification, 0.3 − 0.5 × 10^6^ cells were seeded in the six well plates, cultured in basal media for 24 h, treated with appropriate LA dose and incubated for experimental time as required. Post-treatment, JC1 probe (1.7 μM) was added to the 6-well plate, incubated for 20 min and washed with PBS. Measurements for J-aggregate and monomer were read at wavelength 514/529 and 585/590 nm (Ex/Em) respectively, using BioTek Synergy H1MFG hybrid multi-mode reader (Agilent Technologies, United States). The fluorescence intensity was recorded using monochromator with variable bandwidth. ΔΨm was obtained by calculating the ratio of 520 to 590. Data were expressed as mean ± SD for triplicate.

#### Determination of Cellular Reactive Oxygen Species Level

The ROS generated due to the oxidative stress was quantified by oxidizing 2′, 7′-dichlorofluorescin diacetate (H_2_DCF-DA) into 2′, 7′-dichlorofluorescin. SH-SY5Y cells were treated with the LA at the IC_50_ concentrations for 3 h. Post treatment, DCFH-DA (5 μM) was added and the cells were incubated for 15–30 min at 37°C. Post incubation, cells were observed using Leica DMi8 inverted fluorescence microscope (Leica Microsystems, Germany). The excitation and emission wavelengths were ∼485 and 520 nm respectively. LA blank was used as a negative control. The six well plates were read using BioTek Synergy H1MFG microplate reader (Agilent Technologies, United States). Experiments were performed in triplicate and the data were presented as mean ± SD.

#### Determination of Cell Death by Propidium Iodide

Cell death in LA treated SH-SY5Y cells were confirmed by staining the cells using propidium iodide staining. Briefly, 1.5 mM PI stock solution was prepared in MilliQ water and stored at 2–6°C, protected from light. PI working solution (5 μM) was prepared from the stock solution (1.5 mM) just prior to use. The cells were stained with 5 μM PI and incubated for 5 min, images were acquired using Leica DMi8 inverted fluorescence microscope (Leica Microsystems, Germany). Experiments were performed in triplicate and the data were presented as mean ± SD.

### Molecular Gene Expression Study by Quantitative- Reverse-Transcription PCR

RNA was isolated from cultured SH-SY5Y cells treated with VCO, ECO, and RCO using TRIzol reagent (Thermo Fisher, United States) as per manufacturer’s recommendation. The RNA obtained was stored at −80°C until further use. The RNA yield and purity was calculated by measuring the absorbance at 260 and 280 nm using Synergy™ H1MFG Hybrid Multi-Mode Reader with the Take3 plate micro-volume quantification provision (BioTek Instruments, Inc., United States). The cDNA was synthesized using the previously reported protocols using a cDNA reverse- transcription kit (TaKaRa Bio. Inc., Tokyo) as per the manufacturer’s instructions. Supplementary Table 1 shows the sequences of the primers used in the study. Glyceraldehyde 3-phosphate dehydrogenase (GAPDH) expression was used as an in-house control. Quantitative real-time PCR (qRT-PCR) was carried out using a Biorad CFX Opus 96 Real-Time PCR (Bio-Rad Laboratories, United States) with cDNA as the template at following conditions: PCR − 42 cycles with the following steps, pre-incubation (95°C, 5 min), denaturation (95°C, 10 s), annealing (60°C, 10 s), and elongation (72°C, 10 s). Gene expression was normalized to GAPDH. Fold change was calculated using the 2^–Δ^ ^Δ^ ^cq^ method as described earlier (Balavigneswaran et al., 2020).

### Statistical Analysis

Data was analyzed using the following software’s Microsoft Excel, Graph Pad Prism, MATLAB and R programming running on Windows platform. ANNOVA was used to compute statistical differences in case of three or more groups. Data are represented as mean ± SD.

## Results

### Physio-Chemical Parameters of Virgin Coconut Oil, Crude Coconut Oil, and Refined Coconut Oil

**FIGURE 1:**
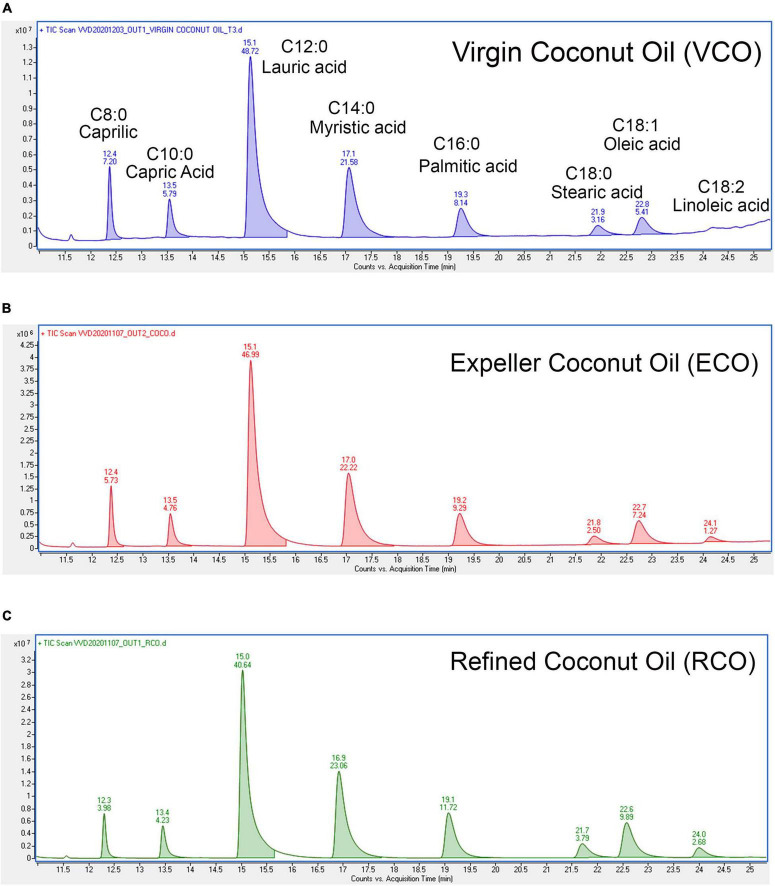
Characterization of fatty acid profile by GC-MS: Total ion chromatogram of **(A)** VCO, **(B)** ECO, and **(C)** RCO, the retention time (RT) was marked on the top of every peak and area sum percentages were marked below the RT in their respective peaks.

The physical parameters of the VCO, ECO, and RCO were studied. The moisture content and volatile matter of the RCO were much lesser than the VCO and ECO. VCO and ECO showed equivalent moisture content and volatile matter (Supplementary Table 2). The decreased amount of moisture and volatile matter was observed in the RCO, due to the evaporation of the volatile substances during the coconut oil refining and solvent extraction process. The density of all the studied coconut oils was 0.906 irrespective of the extraction process. Similarly, there were no major deviations in the refractive index (measured at 40°C) among VCO, ECO, and RCO. However, the color of the ECO (2.70 ± 0.26) was much greater than the RCO (0.67 ± 0.15) and VCO (0.13 ± 0.06). This might be because of the components such as pigments, gums, waxes, trace metals, and other odoriferous volatiles. Of note, the color of the VCO obtained by the cold extraction process is much lower when compared to ECO obtained by the expeller process, at the same time the VCO retains the moisture and volatile matter similar to that of ECO. In general, the increase in additives of the oil increases the hygroscopicity (water-attracting property) of the oil. VCO and ECO had greater hygroscopicity which deeply nourishes and hydrates the skin, therefore it acts as a natural sunscreen (Purnamawati et al., 2017). Since VCO and ECO were not subjected to harsh chemical refining processes such as solvent extraction, it is apparent that they have additional non-glyceride components that could contribute to their moisture and volatile matter. However, it should be noted that the color of VCO was much lower than ECO because the high temperature in the ECO process degrades some of these non-glyceride components leading to an increase in the color of ECO. Further, we studied the chemical parameters of the VCO, ECO, and RCO. The FFA content of the ECO was significantly higher than VCO and RCO (*P* < 0.05) Supplementary Figure 1A. The determination of iodine (IV) was done according to ISO 3961:2018. The IV of the VCO (6.0 ± 0.5) was much lower than the ECO (8.343 ± 0.04) and RCO (8.66 ± 0.5) Supplementary Figure 1B. The higher iodine value of VCO indicates that the double bonds present in the fatty acids of VCO are prone to react with halogens such as iodine compared to ECO and RCO. i.e., VCO has highly saturated fatty acids followed by ECO and RCO, although the unsaturation level of RCO was higher than VCO. There was no significant difference between the IV of ECO and RCO. This was further confirmed from the calculation of individual fatty acids (saturated and unsaturated) relative to the total fatty acid from the total ion chromatogram obtained from GC-MS analysis. The representative total ion chromatogram (TIC) is presented in Figure 1. Then, we evaluated the peroxide value (PV) to measure the oxidation present in the oil, which specifies the content of oxygen as peroxide, especially hydroperoxides. The bar chart Supplementary Figure 1C indicates higher PV in VCO than ECO and RCO. However, the difference among samples was not significant (*P* > 0.05) similarly, there was no significant difference in the saponification value (SV) of VCO, ECO, and RCO (*P* > 0.05) Supplementary Figure 1D. Yet, ECO showed higher value than VCO and RCO. The SV of the VCO, ECO, and RCO were 254.10 ± 3.3, 257.26 ± 0.69, and 254.95 ± 1.17. The results indicated a marginal decrease in the average fatty acid length in RCO and VCO when compared to ECO. In other words, the average molecular weight of all the fatty acids present in the sample as triglycerides present in ECO is comparatively higher that is followed by VCO, and the least mean molecular weight of TG was present in RCO.

### Virgin Coconut Oil and Crude Coconut Oil Have Higher Free Radical Scavenging Capacity Thus Could Be Powerful Antioxidant Source

**FIGURE 2:**
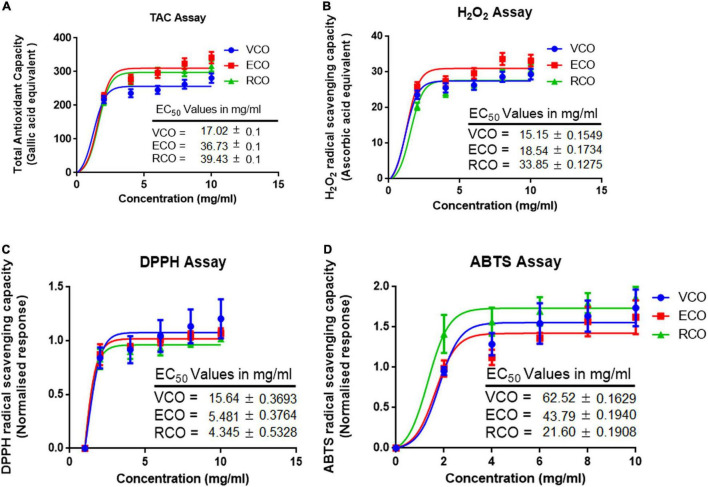
Antioxidant and radical scavenging activity of VCO, ECO, and RCO: **(A)** Total antioxidant capacity **(B)** Hydrogen peroxide scavenging assay **(C)** DPPH radical scavenging assay and **(D)** ABTS radical scavenging assay. All the experiments were performed in triplicate. Data are expressed as mean ± SD (*n* = 3, *p* < 0.05) for all tested dosages (0–10 mg/ml).

Metabolism, inflammation, and other cellular processes generate free radicals in cells. Antioxidants control or obstruct the oxidation of cellular oxidizable substrates by scavenging and blocking the reactive oxygen species (ROS) generation. Total antioxidant activity of the VCO, ECO, and RCO showed a dose-dependent activity increase. We analyzed the results using a non-linear regression model and calculated the half-maximal effective concentration (EC_50_) values. The total antioxidant capacity EC_50_ of VCO (17.02 ± 0.15 mg/ml) and ECO (36.73 ± 0.11 mg/ml) were much higher than the RCO (39.43 ± 0.07 mg/ml) Figure 2A. Similar to total antioxidant capacity, the EC_50_ of H_2_O_2_ scavenging for VCO (15.15 ± 0.15 mg/ml) and ECO (18.54 ± 0.17 mg/ml) were much higher than the RCO (33.85 ± 0.12 mg/ml) Figure 2B. The DPPH free radical scavenging activity of the VCO, ECO, and RCO was shown in Figure 2C. Both VCO and ECO possessed the highest DPPH radical scavenging activity. The EC_50_ of VCO and ECO was 14.80 ± 0.17 and 16.05 ± 0.14 mg/ml. On the other hand, the EC_50_ of RCO was much higher (27.25 ± 0.23 mg/ml) when compared to VCO and ECO. Therefore, we concluded that both the VCO and ECO exhibit similar DPPH free radical scavenging activity. 2,2′-azino-bis(3-ethylbenzothiazoline-6-sulfonic acid) (ABTS• +) radical cation assay complemented DPPH free radical scavenging assay in determining the antioxidant capacity. Figure 2D shows the ABTS radical cation inhibition effect of VCO, ECO, and RCO compared to α-tocopherol. The EC_50_ values of ABTS radical cation inhibition for α-Tocopherol, VCO, ECO, and RCO were 45.90 ± 0.36, 21.60 ± 0.19, 43.79 ± 0.19 and 62.52 ± 0.16 mg/ml, respectively. From the results, it is well observed that VCO exhibit greater antioxidant capacity overall. The EC_50_ values of ECO were much closer to VCO whereas RCO displayed a comparatively lesser antioxidant effect. Overall, our results suggest that VCO and ECO have a powerful antioxidant system to counter the free radicals.

### Virgin Coconut Oil Induces Apoptosis by Lowering the Mitochondrial *Trans-*Membrane Potential (ΔΨm) in Cancer Cells

**FIGURE 3:**
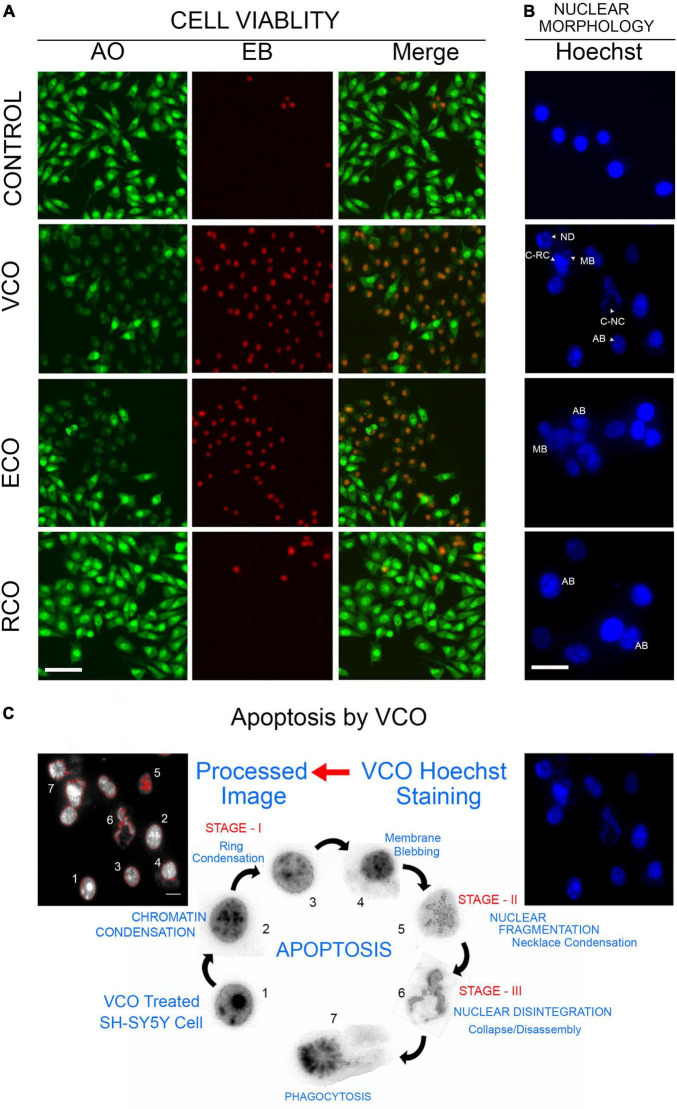
Cellular effect of coconut oil extracted through the various processes in SH-SY5Y cells: SH-SY5Y cells were treated with VCO, ECO, and RCO (0.2 mM). **(A)** AO/EB dual fluorescent staining, green fluorescence indicate live cell and red fluorescence indicates the dead cell population. **(B)** Hoechst fluorescent staining to assess nuclear morphology. **(C)** Detection of morphological changes and apoptosis process in VCO treatment by image processing.

**FIGURE 4:**
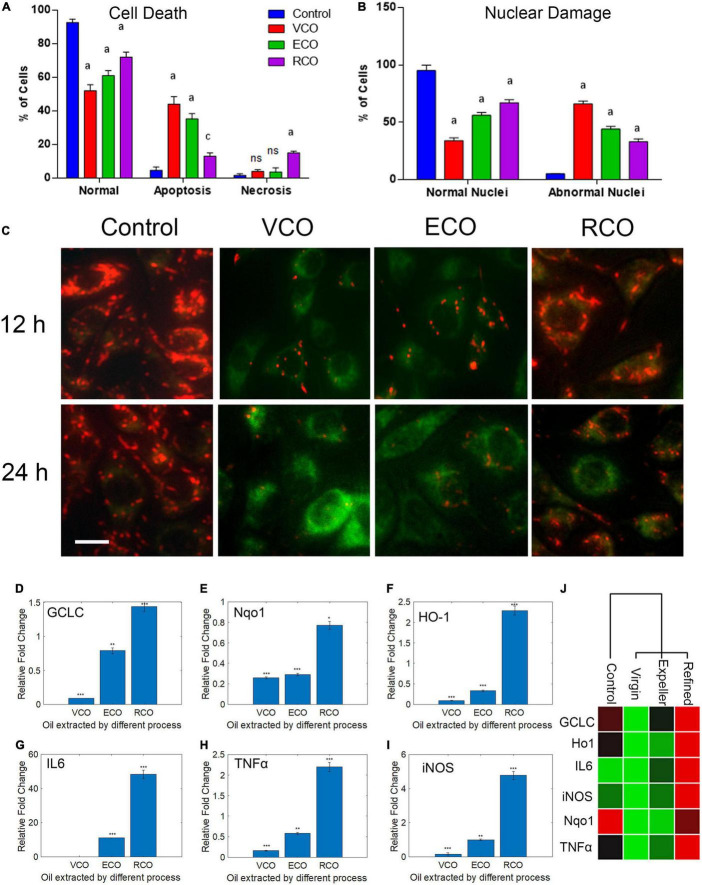
Effect of CO extraction process (VCO, ECO and RCO) on SH-SY5Y cells: **(A)** Bar chart indicating the percentage of normal, apoptosis, and necrotic cells. **(B)** Bar chart indicating the percentage of normal and abnormal nuclei. Triplicate measurements were taken. Data were represented as mean ± SD (^a^*P* < 0.05 and ^c^*P* < 0.001 were considered statistically significant, “ns” represents not significant). **(C)** Determination of mitochondrial *trans-*membrane potential (Δψm). JC- 1 staining of control, VCO, ECO, and RCO treated SH-SY5Y cells incubated for 12 and 24h, post incubation JC1 staining was performed and imaged (scale bar, 10 μm). Figure shows the dose and time dependent effect of oils extracted by various processes on mitochondrial *trans*-membrane potential (Δψm). Differential modulation of antioxidant genes in oil treated SH-SY5Y cells extracted by different processes (VCO, ECO, and RCO) in neuroblastoma cells: **(D)** GCLC, **(E)** Nqo1, **(F)** HO-1 and inflammatory genes, **(G)** IL6, **(H)** TNFα and **(I)** iNOS measured by RT-qPCR. GAPDH was used as the control for PCR experiments. Data represent mean ± SD (*n* = 3); *p < 0.05, **p < 0.001. **(J)** Heat map representing the different expression between groups with ranked lists of genes from high (red) to low (green) in SH-SY5Y cells (*P* < 0.05).

As viability assay revealed, VCO potently inhibits SH-SY5Y cell growth. The morphological changes observed in the treated cells strongly suggest that VCO may have caused apoptosis in cancer cells. AO/EB staining indicates the absence of any significant morphological changes in nuclei and cell membrane integrity of the RCO group. Cell viability is less compromised in RCO treatment whereas ECO and VCO exhibit greater cytotoxicity (Figure 3A). There were certain morphological changes in the VCO and ECO cells such as chromatin condensation, nuclear fragmentation, and disintegration of membrane integrity (Figure 3B). Hoechst immunofluorescent dyes were used to assess the nuclear morphology. Hoechst is a nuclear fluorescent probe, chemically a bisbenzamidazole derivative, which selectively binds to AT in the minor groove of DNA (Ramya et al., 2021). VCO treated SH-SY5Y cells displayed cell death characteristic identical to apoptosis. From the processed image in Figure 3C, it is evident that VCO kills cancer cell by apoptosis process. The chromatin condensation, ring condensation (stage 1 chromatin condensation), membrane blebbing, nuclear fragmentation (stage 2), nuclear disintegration and phagocytosis were clearly observable. Similarly, the nuclear damage was less in RCO treated cells whereas VCO and ECO-treated cells displayed greater nuclear damage consistent with apoptotic cell death. Quantification of the normal, apoptosis, and necrotic cells in VCO, ECO, and RCO treated cell indicate greater apoptosis in VCO followed ECO and a less significant percentage of cells in RCO treatment (Figure 4A). Similarly, quantification of normal and abnormal nuclei percentage indicates significantly greater abnormal nuclei in VCO treated cells, followed by ECO and least in RCO (Figure 4B).

Further, we had determined the mitochondrial membrane potential (ΔΨm) using JC1 fluorescent staining method. Mitochondria play a posh multi-factorial role within the cell. It is documented that the principal role of mitochondria is ATP generation; however, these organelles also are involved in sequestering the calcium (Ca^2+^) or potassium (K^+^) ions and are involved in both generation and detoxification of ROS. It has been reported that during the aging process and in conditions such as neurodegenerative diseases like Alzheimer’s and Parkinson’s there will be minor changes in respiratory chain capacity, substrate supply, glutathione levels, cytoplasmic calcium, and membrane potential (Nicholls, 2002). These functions are interlinked and tightly regulated by the central bioenergetics parameters that include the proton electrochemical gradient across the inner mitochondrial membrane. The mitochondrial membrane potential (ΔΨm) generated by proton pumps (Complexes I, III, and IV) is an important factor in oxidative phosphorylation (Zorova et al., 2018). To make ATP, the hydrogen ions formed from the transmembrane potential (ΔΨm) alongside the proton gradient (ΔpH) are utilized. Under basal conditions, the amount of ΔΨm and ATP are kept relatively stable within the cell. Changes in ΔΨm and ATP could also be deleterious to the cell and should induce cell death. Thus, ΔΨm plays a key role in mitochondrial homeostasis. JC-1 has been reported to be a more reliable indicator of Δψ_m_ (Salvioli et al., 1997). The ratio-metric semi-quantitative assessment of mitochondrial polarization states was performed by monitoring JC-1emission spectra that shifts from green to red. The ratio between red (JC1 aggregates, high ΔΨ_m_) and green (JC1 monomers, low ΔΨ_m_) can be used as a proxy for ΔΨ_m_. In RCO treated cells, ΔΨ_m,_ expressed as red to green FLI ratio was similar to the control cells. ECO treated cells displayed a marginal decrease in ΔΨ_m_ after 12 and 24h. VCO treated cells displayed very low ΔΨ_m_ after 12 and 24h (Figure 4C and Supplementary Figure 2). The reduced ΔΨ_m_ indicate dysfunctional mitochondria conversely the ATP production will be also reduced which will challenge the cancer cell survivability because cancer cells requires a huge supply of ATP than the normal cells (Kim, 2018).

### Virgin Coconut Oil and Crude Coconut Oil Ameliorate Cellular Redox Stress by Modulating the Expression of Key Oxidative Stress Regulatory Genes in SH-SY5Y Cells

The major contributors to the initiation and progression of neurodegenerative diseases such as PD, AD and Schizophrenia are oxidative stress, protein misfolding, and protein aggregation (Bains and Shaw, 1997). In PD, AD, and Schizophrenia a decrease in the levels of the main redox regulator glutathione (GSH) has been observed (Johnson et al., 2012). To assess the redox status in CO supplemented SH-SY5Y, we studied the key genes that respond to oxidative stress *viz* GCLC, HO-1, and Nqo1. The GSH concentration and GCLC activity are accompanied by elevated reactive oxygen species (ROS) (Feng et al., 2017). GCLC protein (Glutamate—cysteine ligase catalytic subunit) is an enzyme encoded by the GCLC gene in humans. GCLC is the first rate-limiting enzyme of glutathione synthesis. An increase in the levels of GCLC transcript and protein often leads to a decrease in cancer cell proliferation and lower intracellular ROS (Mougiakakos et al., 2012). To monitor the modulation of the GSH:GSSG ratio at the molecular level, we examined the GCLC transcript level in VCO, ECO, and RCO treated cells. The level of GCLC was significantly downregulated in VCO (*P* < 0.0001) compared to ECO and RCO. The ECO had significantly lower GCLC transcript levels than the control (*P* < 0.001), whereas the levels of GCLC were higher in RCO treated SH-SY5Y cells (*P* < 0.0001) (Figure 4D). This indicates the formation of the cellular antioxidant glutathione (GSH) was not triggered upon cellular uptake of VCO and ECO. However, RCO supplementation triggers the cellular antioxidant response genes. Further, we studied the modulation of Nqo1 expression in VCO, ECO, and RCO treated SH-SY5Y cells. Nqo1 transcript levels were significantly lower in VCO treated cells followed by ECO and RCO (Figure 4E). Interestingly, HO-1 is significantly increased (*P* < 0.0001) in the RCO treated SH-SY5Y cells corroborating with the GCLC levels (Figure 4F). The levels of HO1 were significantly decreased in VCO and ECO-treated cells (*P* < 0.0001). Of note, HO-1 over-expression protects cells from glutamate toxicity and H_2_O_2_-induced cell death (Chen et al., 2000).

This indicated that the RCO treatment possibly increases the generation of free radicals. Nqo1 is induced in oxidative stress to stabilize and preserve the levels of critical proteins against degradation (Ross and Siegel, 2018). Nqo1 exhibits its protective role by reducing a wide array of substrates such as ubiquinone and vitamin E quinone to their corresponding antioxidant forms. One-electron reduction of quinone leads to the production of radical species, whereas Nqo1 prevents the process enzymatically (Ross and Siegel, 2017). Therefore, higher Nqo1 gene expression is an indicator for oxidative cellular response damage control event. From the results, it is well observed that associated external antioxidant compounds in the VCO and ECO are also responsible for the beneficial effects. RCO is devoid of these external antioxidant compounds therefore the inherent cell antioxidant defense mechanisms are brought into the play. Overall, the excessive ROS produced by the cell’s inherent mechanism and through β-oxidation of fatty acids are neutralized by additional antioxidant molecules present in VCO and ECO conversely RCO treated cells are dependent on the inherent cellular antioxidant defense mechanism to neutralize these excessive ROS. Although significantly down regulated, compared to GCLC and HO-1 there is a marginal increase in the Nqo1 gene expression in VCO treatment. The primary reason is that both VCO and ECO contain antioxidant molecules that include a wide range of quinones. It is worthy to mention that VCO is also rich in α-tocopherol content that is a known Nqo1 inducer (Feng et al., 2010). The FA inside the cells is broken down to produce energy by the fatty acid β-oxidation process. In each round of β-oxidation, one molecule of acetyl-CoA (which enters the TCA cycle), one molecule of NADH, and one molecule of FADH_2_ are produced. The electron transport chain uses the NADH and FADH_2_ produced by both β-oxidation and the TCA cycle to produce ATP. It is interesting to note that the ability of Nqo-1 to protect target proteins from 20S proteolytic cleavage is dependent upon the addition of NADH. In addition, Nqo1 is a FAD-binding protein that reduces quinones to hydroquinones by forming homodimer. Altogether, the presence of α-tocopherol as primary inducer and availability of NADH and FAD in excess inside the cell have a synergistic effect on exhibiting the protective effect by VCO and a marginal increase in Nqo-1 transcript levels. Other than Nqo1, both GCLC and HO-1 levels were extremely down regulated in VCO treatment.

### Virgin Coconut Oil and Crude Coconut Oil Treatment Play a Pivotal Role in Cytokine Regulation and Inflammatory Response

To investigate any potential contribution of VCO, ECO, and RCO treatment in cytokine regulation and inflammatory response, we treated the SH-SY5Y cells with VCO/ECO/RCO and examined its effect on RNA expression of IL6, TNFα, and iNOS by qRT-PCR. The VCO, ECO, and RCO treatment differentially modulated the expression of IL6 levels. VCO treatment down-regulated IL6 (0.06 fold, *P* < 0.0001) whereas RCO treatment up-regulated IL6 up to 48.34 fold (*P* < 0.0001). ECO treatment did moderately up-regulated IL6 upto 11 fold (*P* < 0.0001) when compared to control (Figure 4G). The same trend was followed for TNFα, where VCO and ECO down-regulated TNFα by 0.16 fold (*P* < 0.001) and 0.58 fold (*P* < 0.0001) respectively, whereas RCO treatment significantly up-regulated TNFα transcript level by 2.2 fold (*P* < 0.0001) (Figure 4H). Of note, IL-6 is a proinflammatory cytokines that is responsible for the activation of immune response. IL6 is a key cytokine that plays a vital role in hormonal balance and maintaining the free fatty acid levels. Upon VCO treatment, IL6 was significantly down regulated indicating the no major deviation in cell homeostasis. Although, the cancer cells undergo apoptosis process, there is no inflammatory response. Macrophages tightly regulate the clearance of the damaged cells in a programmed fashion through apoptosis. On the other hand, Macrophages and lymphocytes secrete TNFα in response to cell damage. Our observation is concomitant to previous studies where IL6 and TNFα were found to be elevated in patients with lipid abnormalities and insulin resistance (Pickup et al., 1997; Bahceci et al., 2007; Popko et al., 2010; Fontanella et al., 2021).

Further, the highest upregulation of iNOS was observed for RCO treated cells (4.27 fold, *P* < 0.0001). ECO treatment showed no major deviation when compared to control cells (*P* > 0.05) (Figure 4I). Summarizing, VCO and ECO-treated cells had down-regulated all the investigated inflammatory genes viz IL6, TNFα, and iNOS. On the contrary, RCO treatment had upregulated all the investigated inflammatory genes (Figure 4J). Taken together, our analysis demonstrates RCO treatment induces the expression of cytokines and inflammatory response genes in SH-SY5Y cells probably putting the neural cells under inflammatory conditions (Zhao et al., 2010). It is apparent from the results that VCO offers a greater beneficial effect in protecting the cells. RCO contributes to inflammation in neural cells that would eventually lead to neurodegeneration as in the case of AD and PD. Therefore, we strongly suggest to include either VCO or ECO in diet and exclude RCO (those coconut oil that are extracted by solvent refining process) from the diet especially for growing children and old-aged people.

### Lauric Acid Induces Reactive Oxygen Species Mediated Apoptosis by Capsizing the Mitochondrial Membrane Potential (ΔΨm) in SH-SY5Y Cells

**FIGURE 5:**
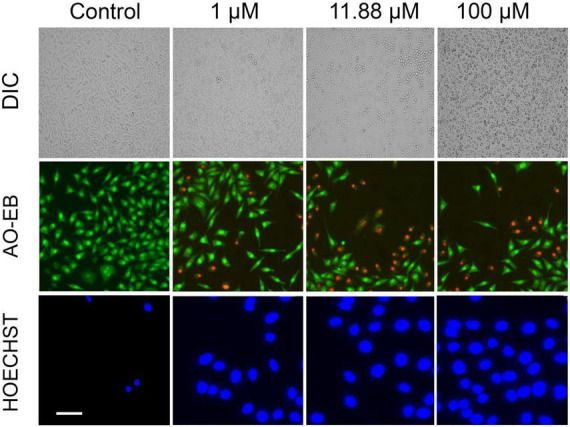
Effect of LA on SH-SY5Y cells: Photomicrograph showing the cytotoxic effect of LA in dose dependent manner (horizontal lane): Panel 1: Differential Interference Contrast (DIC) images of LA treated cells (scale bar, 50 μm). Panel 2: Acridine Orange/Ethidium Bromide (AO-EB) dual fluorescent staining of LA treated cells (scale bar, 20 μm). Panel 3: Nuclear staining using Hoechst immunofluorescent dye after 24 h of respective LA treatment (scale bar, 10 μm).

Further, we questioned the reason behind the beneficial effect of VCO and ECO over RCO. Profiling the fatty acid using GC-MS revealed the greater lauric acid (LA) content in VCO (other than the non-glyceride components that are present in the VCO). Results indicated the occurrence of 48.72% LA (C12:0) in VCO. ECO and RCO had 46.99 and 40.64% LA, respectively, relative to the total fatty acid Supplementary Figure 1E. LA content was significantly higher in the VCO, followed by ECO and RCO. To understand the cellular and molecular effect of LA in SH-SY5Y cells, we studied the role of LA in ameliorating the cellular redox state. We hypothesized that LA intervention will bring the cellular redox threshold of SH-SY5Y neuroblastoma cells closer to the normal cells; thereby it induces ROS-mediated apoptosis. To validate the hypothesis, initially, an MTT assay was performed to study the cytotoxic effect of the LA in the neuroblastoma cell lines (SH-SY5Y cells). Increasing concentrations of LA were treated in SH-SY5Y for 24, 48, and 72 h. A significant time and dose-dependent effect were observed in SH-SY5Y cells at micromolar concentrations. The IC_50_ value of LA was calculated as 11.88 ± 2 μM based on the MTT assay (Supplementary Figure 2A). Further, an acridine orange/ethidium bromide (AO-EB) dual staining assay was performed to investigate the morphological changes and cytotoxicity. SH-SY5Y cells were treated with 1 μM, 11.88 μM (IC_50_ concentration), and 100 μM LA for 24 h, stained for AO-EB, and analyzed by fluorescent microscopy. Results indicate a dose-dependent increase in cytotoxicity (Figure 5). LA treatment in SH-SY5Y cells showed characteristic apoptotic features such as severe membrane blebbing, cytoplasmic vacuolation, and the presence of apoptotic bodies. 1 and 11.88 μM LA treatment revealed comparatively lesser cells with abnormal nuclear morphologies such as chromatin fragmentation, bi-nucleation, and nuclear fragmentation when compared to the 100 μM LA. In addition, marginalization of chromatin was also observed in the 11.88 and 100 μM LA treated SH-SY5Y cells. Furthermore, late apoptosis was observed by the indication of dot-like chromatin and innumerable micronuclei in cells. The results indicated that 11.88 and 100 μM LA treatment kills the neuroblastoma cells by apoptosis.

**FIGURE 6:**
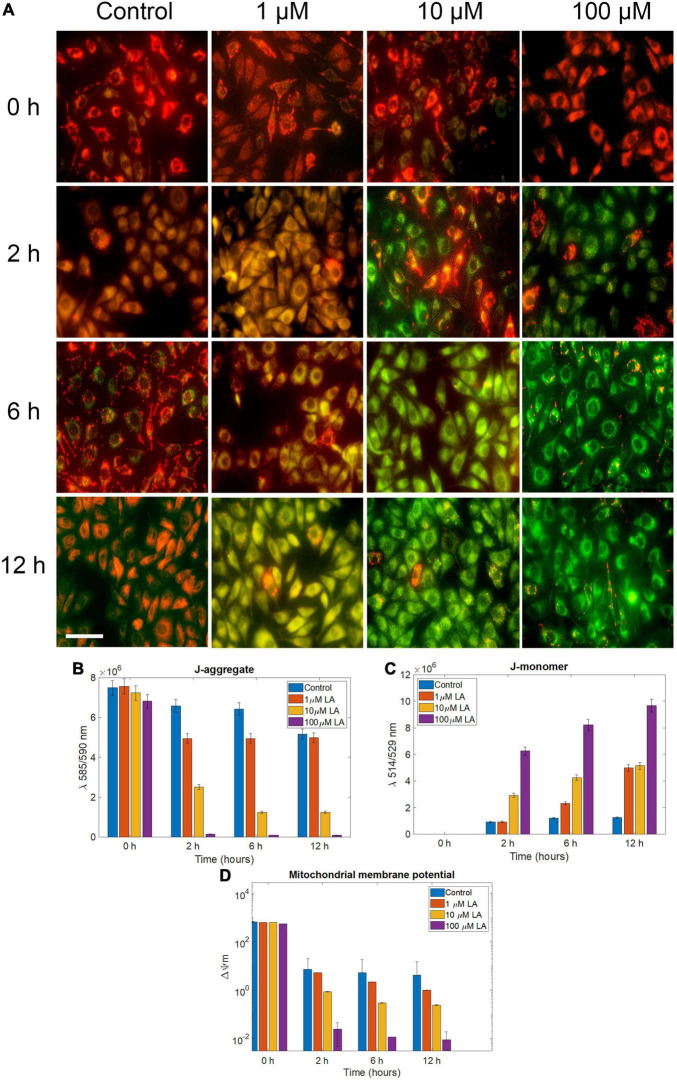
Effect of LA on mitochondrial *trans-*membrane potential (Δψm): **(A)** JC- 1 staining of 0, 1, 10, and 100 μM LA treated SH-SY5Y cells incubated for 0, 2, 6 and 12 h to reflect the dose and time dependent effect of LA (scale bar, 50 μm). **(B)** J-aggregate, normalized fluorescence intensity measured at λ590. **(C)** J-aggregate, normalized fluorescence intensity measured at λ520. **(D)** Mitochondrial *trans-*membrane potential (Δψm) calculated by a decrease in the λ590/520 ratio. Data represent mean ± SE.

Based on the observations, we concluded that LA treatment induces apoptosis in SH-SY5Y cells. However, to determine whether the effect was induced by ROS production, we determined the mitochondrial membrane potential (ΔΨm) using JC1, probed the cellular ROS using H_2_DCF-DA, and determined the cell death using propidium iodide (PI). In 1 and 10 μM LA (dose closer to LA IC_50_ in SH-SY5Y, 11.88 μM) treated cells, ΔΨ_m_, (expressed as red to green FLI ratio) was similar to control cells until 2 h. However, after 2 h, 10 and 100 μM LA treated cells had started to exhibit low ΔΨ_m_ (Figures 6A–D). The reduced ΔΨ_m_ indicates dysfunctional mitochondria. Conversely, the ATP production is going to be also reduced which will have a severe blow to the survival of cancer cells, which need an enormous supply of ATP than the normal cells (Kim, 2018; Gilbert et al., 2019). Of note, ΔΨ_m_ depends majorly on the inner membrane integrity and maybe impaired by ROS generation. It is apparent from our results that the ΔΨ_m_ is lowered upon LA intervention and the inner membrane integrity was distorted within 6 h after exposure to 10 and 100 μM LA. Lower concentration of LA such as 1 μM did not affect the ΔΨ_m_ (Figure 6D).

**FIGURE 7:**
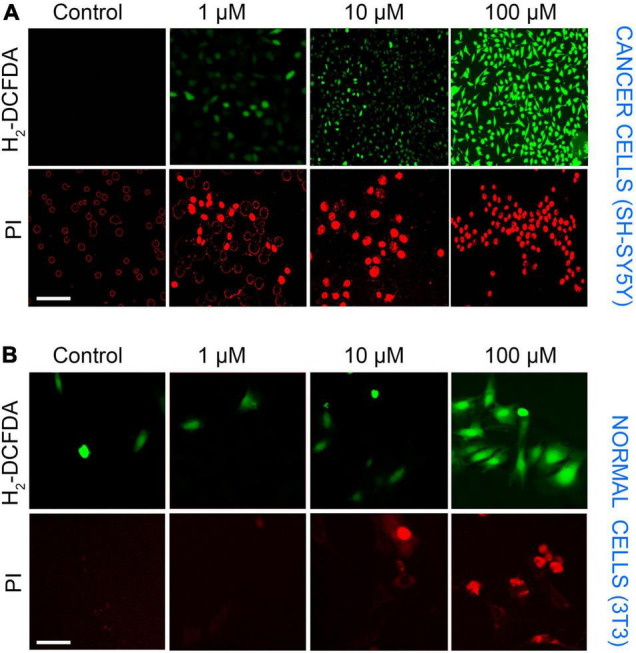
SH-SY5Y cells undergo ROS-mediated apoptosis upon LA treatment in cancer and normal cells. **(A)** Top panel—ROS production by H_2_DCFDA (scale bar, 50 μm). SH-SY5Y cells were incubated with LA (0, 1, 10, and 100 μM) for 3 h and stained with H_2_DCFDA. Bottom panel—Propidium iodide (scale bar, 20 μm). **(B)** Top panel–ROS production by H_2_DCFDA (scale bar, 20 μm). 3T3 cells were incubated with LA (0, 1, 10, and 100 μM) for 3 h and stained with H_2_DCFDA. Bottom panel—Propidium iodide (scale bar, 20 μm).

**FIGURE 8:**
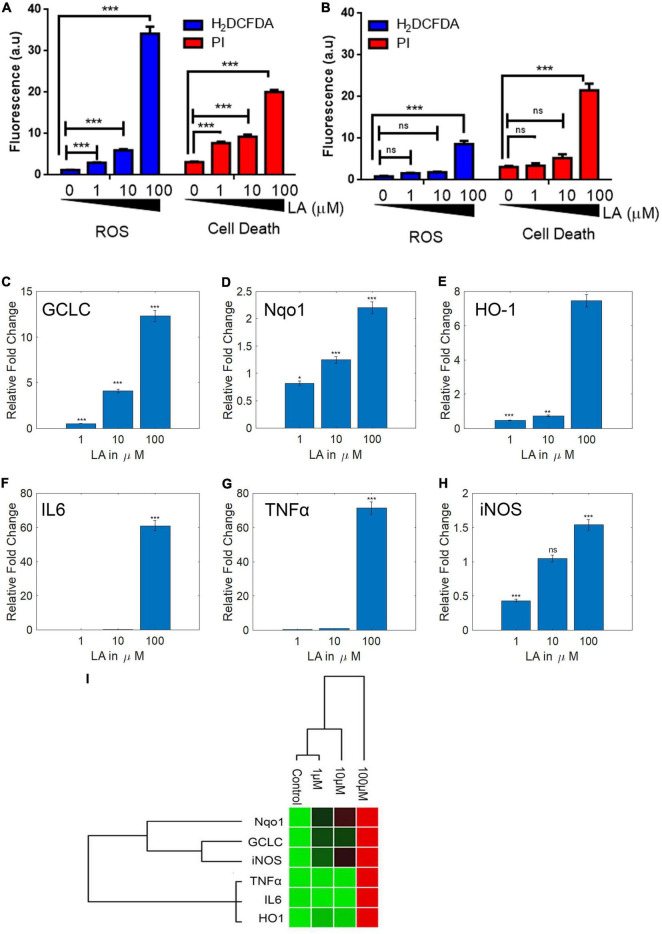
Lauric acid (LA) induces ROS-mediated apoptosis in SH-SY5Y cells but 3T3 cells are less affected. Bar chart indicating the cellular ROS production **(A)** proportionate to cell death **(B)** at varying LA concentrations. LA modulates antioxidant and inflammatory genes differentially. SH-SY5Y cells were treated with LA (0, 1, 10, and 100 μg/mL), total RNA was isolated, reverse transcribed to cDNA and analyzed by qPCR for expression of antioxidant genes GCLC **(C)**, HO-1 **(D)**, Nqo1 **(E)**, and anti-inflammatory genes IL6 **(F)**, TNFα **(G)**, iNOS **(H)**. **(I)** Gene expression profiling of LA mediated transcript modulation. Each experiment was repeated at least with three parallel replicates. GAPDH was used as the control. Data represent mean ± SD (*n* = 3); *p < 0.05, **p < 0.001, ***p < 0.0001.

The presence of ROS in excess inside the cells causes oxidative stress, mitochondrial dysfunction, and forces the cell to undergo apoptosis. LA impaired the mitochondria’s inner membrane integrity (low ΔΨ_m_), the effect could likely result from the excessive ROS generation by mitochondria. Therefore, we assessed the ROS production in SH-SY5Y cells using an H_2_DCF-DA fluorescence probe. The generation of ROS by LA was increased in a dose-dependent manner (Figure 7A). We found that 1 μM LA did not affect ROS production. However, treatment with 10 μM LA markedly increased the intracellular ROS level in SH-SY5Y cells. LA treatment at higher concentration (100 μM) dramatically increased the ROS production. Further, to verify the cell death in SH-SY5Y cells, we used propidium iodide (PI) after LA treatment. As shown in Figure 7B bottom panel, LA (100 μM) increased cell death proportionate to the ROS generation, whereas 1 μM LA generated ROS to a lesser extent and far less compared to 10 and 100 μM LA. Normal 3T3 cells showed relatively lesser ROS production and cell death (Figure 7B). However, when 3T3 cells were treated with 100 μM LA, the ROS production and cell death were comparatively higher than the control 3T3 cells (Figure 8B), yet much lower than the SH-SY5Y cells (Figure 8A). Taken together, presumably LA treatment induces ROS-mediated apoptosis and cell death in SH-SY5Y cells. The results indicate that LA treatment increases ROS production, induces oxidative stress, lowers the ΔΨ_m,_ and leads to cell death in neuroblastoma cells.

### Lauric Acid Alters Cellular Redox Homeostasis and Modulates Key Inflammatory Gene Expression in SH-SY5Y Neuroblastoma Cells

Reactive oxygen species plays a vital role in neuroinflammation and neurodegeneration (Mittal et al., 2014). Earlier we had shown that the VCO treatment had significantly down-regulated the antioxidant genes (GCLC, Nqo1, HO1) and inflammatory gene (IL6, TNFα, and iNOS) expression in SH-SY5Y cells. Since LA is the major fatty acid in VCO, we hypothesized that LA treatment may down-regulate the expression of antioxidant and inflammatory genes in a dose-dependent manner. To understand the molecular effect upon LA exposure, we studied the central antioxidant and inflammatory genes. LA treatment altered the expression of key cellular redox enzymes such as GCLC, HO-1, and Nqo-1. As opposed to our hypothesis, the RNA expression levels of GCLC, HO-1, and Nqo-1 were increased in a dose-dependent manner including the dose at IC_50_ concentration (Figures 8C–E). The levels of GCLC were downregulated in 1 μM LA treated cells (0.52 fold, *P* < 0.001), whereas 10 and 100 μM LA treatment upregulated the expression of GCLC to 4.11 and 12.30 fold, respectively (*P* < 0.001). Similarly, the levels of Nqo1 were marginally downregulated in 1 μM treated LA upto 0.82 fold (*P* < 0.01), whereas the same were upregulated in 10 and 100 μM LA treated cells upto 1.25 and 2.20 fold, respectively (*P* < 0.01). HO-1 was significantly downregulated in both 1 and 10 μM treated SH-SY5Y cells (0.47 and 0.75 folds respectively) and the same was up-regulated unto 7.46 fold in 100 μM LA treated cells.

Furthermore, we studied the modulation of inflammatory genes upon LA treatment in SH-SY5Y cells. IL6 along with other inflammatory genes in SH-SY5Y cells were significantly down regulated at 1 μM (0.09 fold, *P* < 0.0001) and 10 μM LA treatment (0.14 fold, *P* < 0.0001). On the other hand, 100 μM LA treatment significantly upregulated the levels of IL6 transcript (60.97 fold, *P* < 0.0001) (Figure 8F). The transcript levels of TNFα were downregulated in 1 μM (0.53 fold, *P* < 0.0001) and 10 μM LA treatment (0.89 fold, *P* < 0.0001). 100 μM LA upregulated TNFα transcript levels to 71.51 fold compared to control (Figure 8G). Similarly, iNOS was downregulated in 1 μM LA treatment (0.43 fold, *P* < 0.001). 10 μM LA treatment did not affect the iNOS transcript level when compared to control (1.05 fold). However, 100 μM LA treatment increased the iNOS transcript level 1.5 fold higher when compared to control (*P* < 0.5) (Figure 8H). From the results, it can be observed that 1 and 10 μM LA concentration primarily confers the cytoprotective effect by activating the GCLC, Nqo1, and HO1 genes and doesn’t trigger the inflammatory pathway genes IL6, TNFα, and iNOS similar to VCO (Figure 8I). Whereas, 100 μM LA triggers the activation of IL6 mediated inflammatory response pathway. The reason behind the observation is the presence of powerful antioxidant system in VCO, the non-triglyceride antioxidant molecules in VCO cluster counters any ROS generated by β-oxidation of fatty acids and other cellular processes. On the contrary treatment with LA lack the advantage of additional support from external antioxidant molecules to counter the cellular ROS therefore to balance the cellular GSH:GSSG ratio and to maintain the cellular redox balance, the cells had up-regulated GCLC and Nqo1. This observation indicates that LA treatment at IC_50_ concentration in SH-SY5Y cells maintains the cellular redox balance without triggering the inflammatory response. However, at a very high concentration such as 100 μM LA, inflammatory pathway genes are triggered that may tend the cell to undergo apoptosis, this could be primarily due to the accumulation of excessive ROS inside the cell because of mitochondria membrane depolarization (low ΔΨm) (Supplementary Figures 3A–C).

### Lauric Acid at Physiological Concentration Ameliorates Neuro-Inflammation in Dose Dependent Manner

Despite an exhaustive literature search, we were unable to find the concentration of circulating plasma LA concentration neither in normal human subjects nor in pathological conditions or in any animal models. However, Kaska et al. (2014) had reported the percentage of LA in morbidly obese patients after maintaining a low calorie diet for 3 months as approximately 1% (relative to total FA), whereas, in the same study the percentage of palmitic acid (C16:0) was reported as 24.5% (relative to total FA) (Kaska et al., 2014). However, the absolute values were not provided. Therefore we calculated the ratio of lauric to palmitic according to Kaska et al. (2014) that would be approximately 1:24.5 (i.e., for every 1,000 μM PA apparently there should be 40.81 μM LA). We know that the normal physiological concentration of PA (C16:0) would be ranging from 0.3 to 4.1 mmol/L. Considering the 1:24.5 ratio for LA to PA derived from Kaska et al. (2014) then the physiological concentration of LA should be around 0.12–0.164 mmol/L (i.e.) 12–164 μM. However, this has to be validated by further studies. Of note, we had observed the IC_50_ of LA in SH-SY5Y cells as 11.88 μM which is closer to the lower limit.

**FIGURE 9:**
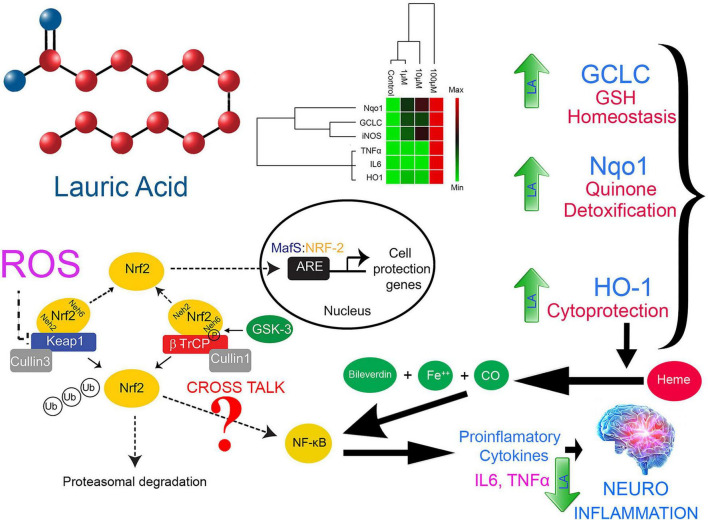
Proposed model for LA-mediated cellular redox threshold balancing, ROS induction, inflammatory signaling and its role in neuro-inflammation

A dose-dependent increase in the productions of IL6, TNFα, and iNOS at physiological concentration (10 μM) together with ROS production in cells treated with LA allowed us to hypothesize the involvement of Nrf2-ARE pathway genes in the apoptosis process (Figure 9). However, at higher concentration (100 μM), LA induces cytokine storm by hyperactivating IL6 and TNFα. Under oxidative stress, the Nrf2 protein dissociates from Keap1 and translocates into the nucleus, where it *trans*-activates the antioxidant genes that contain the antioxidant response element (ARE), 5′-TGAG/CnnnGC-3′ in their promoters. This include NAD(P)H:quinoneoxidoreductase (Nqo1), and heme oxygenase-1 (HO-1) (Mittal et al., 2014). On the other hand, the production of interleukin-6 (IL6) is the hallmark of NF-κB activation, whose role as an anti-inflammatory and pro-inflammatory signal is still controversial. The eventual outcome of NF-κB signaling is the activation of inflammatory genes. From our results, we can interpret that LA induced the non-canonical activation of the NF-κB signaling pathway mediated by intracellular ROS. Previous reports indicate that the intracellular ROS function as a second messenger and induces the phosphorylation of Rel-A at Ser-276 residue. This pathway is dependent on the activation of the catalytic subunit of protein kinase A (PKAc) by TNFα (Brasier, 2010). Accordingly, TNFα-induces the RelA Ser-276 phosphorylation. Our results showed that that LA supplementation induces the expression of both IL6 and TNFα in a dose-dependent manner. Taken together, it is implicit that LA supplementation activates the NF-κB-dependent gene expression. Here we show, LA orchestrates the cytokines and inflammatory pathway at the transcriptional level and promotes apoptosis. This lays a strong foundation for probing the epigenetic regulatory aspects of LA in the future. Further studies are needed to examine the effect of LA on expression and epigenetic control on inflammatory genes and epigenetic factors on transcriptional regulation.

### Lauric Acid did Not Affect Normal Cells

To validate our pathway prediction of dose-dependent activity of neuroprotection, we extended our study to evaluate their potential in normal cell. Because the redox threshold in normal cells will be multi-fold lower than the cancer cells. A relatively low ROS level acts as a second messenger that aids in cell proliferation and differentiation. However, if the ROS levels are higher than the threshold then they warrant apoptosis and cell death. Thus, mitochondria wangle the cell death depending on the cellular redox balance (ROS), and the expression of proapoptotic factors, among others. The L929 and 3T3 cells were taken as a model and treated with higher concentration (100 μM) of LA. We further studied their structural change on various time intervals. The IC_50_ of LA in L929 cells was 349.9 μM (Supplementary Figures 2C,D). The cells were unaffected upon treatment, and interestingly an increase in cell proliferation was observed in 3T3 cells (data not shown). The results indicate that LA did not affect the normal cells.

### Cellular Redox Nexus of Lauric Acid in Inflammation, Reactive Oxygen Species Release, and Apoptosis

Cancer cells meet their ATP demand from mitochondria. Of note, mitochondria are the chief site of ROS production, also the epicenter for autophagy and cell death. In abnormal circumstances such as cancer, infection, or derailed cell cycle, the ATP demand will be higher, which eventually forces the cell to synthesize ATP at a higher rate, which would result in excessive ROS generation (El-Shaqanqery et al., 2021). The higher redox threshold of cancer cells indicates that mitochondria acclimatize to higher ROS concentration. Our results indicate LA intervention lowers the ΔΨm in SH-SY5Y cells (Supplementary Figure 3C). Conversely, the ATP production is going to be reduced because harnessing of ΔΨm is important to create the torque that is used by ATP synthase during ATP synthesis. Reduced ATP concentration will affect the survivability of cancer cells (Kim, 2018). Under basal conditions, the ATP concentration in the intracellular component will be around 1–10 mM with ATP to ADP ratio of 1,000 and in the extracellular compartment the concentration will be around 10–100 nM. Further, the estimated rate of transmembrane proton flux through mitochondria ATP synthase is 3 × 10^21^ protons per second, thus the rate of recycling of ADP to ATP would be around 9 × 10^20^ molecules/sec. Moreover, the concentration of the extracellular ATP in the cancer cells are very high > 100 μM (Gilbert et al., 2019). The lowering of ΔΨ_m_ significantly reduces the rate of ADP recycling. Furthermore, it reduces the ATP/ADP ratio in the internal compartment, thus altering the balance in ATP concentration between the internal and external compartment. In addition, nitric oxide (NO) production is often associated with inflammation and *vice versa* (iNOS produces large amounts of NO as a defense mechanism). NO competes with oxygen to bind with cytochrome oxidase heme active site. An increased NO will result in successful competition with molecular oxygen, resulting in decreased binding efficiency of oxygen that ultimately results in the reduced proton electrochemical gradient thus interrupting the ATP synthesis. The reduced ΔΨ_m_ coupled with increased intracellular NO concentration reduces the ATP production significantly, triggering ROS mediated inflammatory response and apoptosis, resulting in cancer cell death by apoptosis (Walford et al., 2004).

**FIGURE 10:**
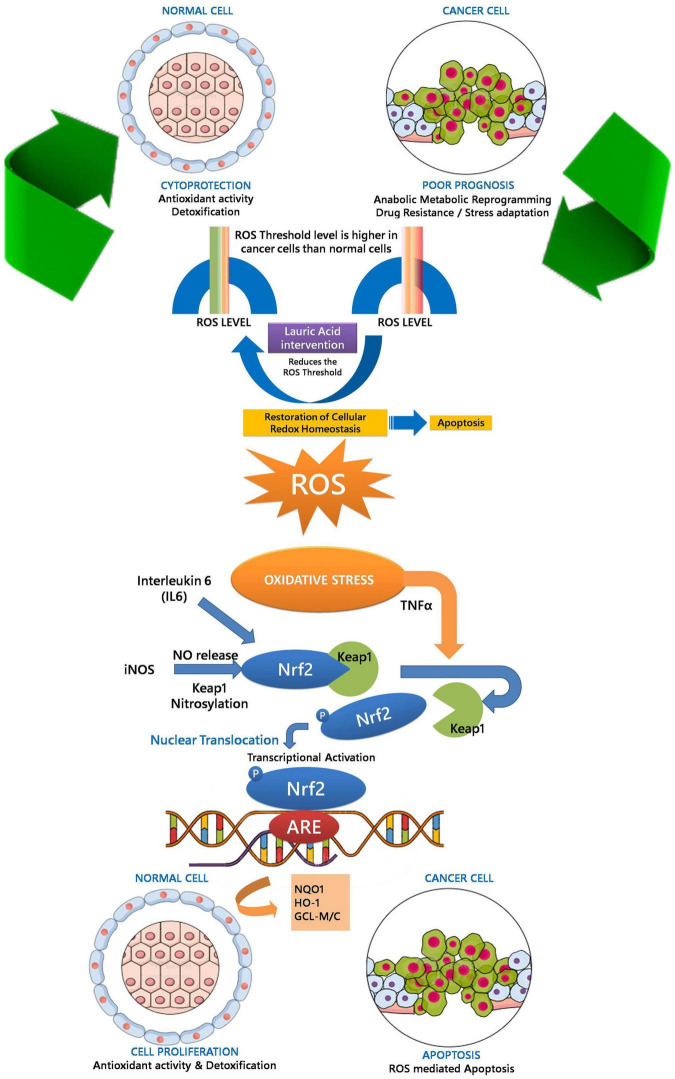
Oxidative stress mediated inflammation development and progression-involving activation of Nrf2-Keep1-ARE signaling module facilitating ROS mediated apoptosis in neuroinflammation ultimately leading to neurodegeneration.

Lipids have a central role in offering fatty acids to fulfill the high-energy requirements of the cancer cells. Understanding the metabolic switch during tumorigenesis and lipid availability had gained recent attention. With the Warburg effect and the increased glutaminolysis, lipid metabolism transpires as an essential for tumor development, survival, and progression (Fernández et al., 2020). Interestingly, SH-SY5Y used in the present study confers two different perspectives. The SH-SY5Y cells are proliferative and more uniform model in studying the PD and AD (Xicoy et al., 2017). Herein we had used them to understand the inflammatory response and neurodegeneration that mimics in PD and AD (Simone et al., 2021). In addition, SH-SY5Y cell line was derived from a neuroblastoma patient bone marrow and the cells have cancerous properties. As a result, the cancerous nature of the cells influences their viability, differentiation fate, metabolic properties, and genomic stability (Xicoy et al., 2017). We had exploited the cancerous nature to study apoptosis and response of LA in tumor microenvironment. In the present manuscript, we show the increase in ROS level in SHSY5Y cells upon LA treatment. Our results constitute the up-regulation of IL6, iNOS, and TNFα in SH-SY5Y cells upon LA treatment in a dose-dependent manner. However, the normal cells were healthy and not affected depending upon the concentration. Based on our observation, we postulate that LA induces oxidative stress mediated inflammation in cancer cells. The oxidative stress mediated inflammation development and progression involves the activation of the Nrf2-Keep1-ARE signaling module. The Keap1/Nrf2/ARE signaling pathway regulates anti-inflammatory genes. Under basal conditions, Nrf2 is concealed with Keap1. LA intervention maintains the ROS level within the redox threshold that is much lower for normal cells. In SH-SY5Y neuroblastoma (cancer) cells, the redox threshold will be much higher than the normal cells. Excessive ROS presence in the cellular milieu puts the cell under oxidative stress. However, LA intervention reduces the redox threshold of cancer cells closer to the normal cells and triggers an inflammatory response through IL6 signaling. This event may call for translocation of Nrf2 to nucleus by dissociating themselves from Keap1. The nuclear translocation of the Nrf2 activates the ARE-gene cluster (including GCLC, Nqo1, and HO-1) facilitating ROS-mediated apoptosis in neuroinflammation ultimately leading to neurodegeneration and cell death in cancer cells. On the contrary, the beneficial effect of GCLC, Nqo1, and HO-1 will help in cell proliferation in normal cells. The overall proposed model was depicted in Figure 10. On the other hand, IL6 expression induces the activation of the NF-κB pathway. Therefore, crosstalk between Nrf2 and NF-κB pathway was inevitable in maintaining the cellular redox balance mediated by LA. However, the neuroprotection mechanism of LA along with other molecules in VCO is yet to be studied in detail. The composite mixture may give a clear picture.

## Conclusion

In the present manuscript, we had shown that LA orchestrates the cytokines and inflammatory pathway at the transcriptional level. The cellular redox nexus involves the cross talk of Nrf2 and the NF-κB pathway. Inflammation signaling plays a pivotal role in regulating the cellular redox state and deciding the cell fate. This lays a strong foundation for probing the epigenetic regulatory aspects of LA in the future. Further studies are needed to examine the effect of LA on expression and epigenetic control on inflammatory genes and elongation factors on RNA processing. In conclusion, dietary regulation of lipids, fatty acids, and subsequent cellular redox threshold altercation will represent a fruitful ground for understanding cellular ROS behavior, cell survival, inflammation, and cancer. Yet, studying the interaction of LA with other molecules present in the VCO may give a detailed picture of how VCO being highly efficient for the infants and old-aged people.

## Data Availability Statement

The original contributions presented in the study are included in the article/Supplementary Material, further inquiries can be directed to the corresponding author/s.

## Author Contributions

VR and BK designed the experiments. VR performed the experiments. EK and CB verified the results independently. VR and KS wrote the first draft of the manuscript. VR, CB, and KS analyzed data and wrote sections of the manuscript. BK supervised the project, involved in all the activities, finalized the final version of the manuscript, and approved the final draft. All the authors contributed to manuscript revision, read, and approved the submitted version.

## Conflict of Interest

KS and EK were employed by V.V.D and Sons Private Limited, Thoothukudi. The remaining authors declare that the research was conducted in the absence of any commercial or financial relationships that could be construed as a potential conflict of interest. The handling editor declared a past collaboration with one of the author CB.

## Publisher’s Note

All claims expressed in this article are solely those of the authors and do not necessarily represent those of their affiliated organizations, or those of the publisher, the editors and the reviewers. Any product that may be evaluated in this article, or claim that may be made by its manufacturer, is not guaranteed or endorsed by the publisher.

## Supplementary Material

Supplementary Figure 1 Chemical parameters of VCO, ECO, and RCO. **(A)** Free Fatty Acid **(B)** Iodine Value **(C)** Peroxide Value **(D)** Saponification Value **(E)** Percentage Fatty acid composition determined using GC-MS relative to total fatty acid in VCO, ECO, and RCO. Data represent mean ± SD.

Supplementary Figure 2 Assessment of cytotoxicity of LA in cancer (SH-SY5Y) and normal (L929) cells. **(A)** Cytotoxicity of LA in cancer (SH-SY5Y) cells by MTT assay. **(B)** and photomicrographs showing LA treated SH-SY5Y cells, panel 1—DIC, panel 2–AO-EB staining (scale bar, 50 μm), **(C)** Cytotoxicity of LA in normal (L929) cells by MTT assay **(D)** photomicrographs showing LA treated L929 cells, panel 1—DIC, panel 2–AO-EB staining (scale bar, 100 μm). Data represent mean ± SD.

Supplementary Figure 3 JC-1 analysis of mitochondrial membrane potential in SH-SY5Y cells determined after 12 and 24h. **(A)** J-aggregate, normalized fluorescence intensity measured at λ590. **(B)** J-aggregate, normalized fluorescence intensity measured at λ520. **(C)** Mitochondrial *trans-*membrane potential (Δψm) calculated by a decrease in the λ590/520 ratio. Data represent mean ± SE.
