# Supplementary figures and images for: Dual Roles of Coconut Oil and Its Major Component Lauric Acid on Redox Nexus: Focus on Cytoprotection and Cancer Cell Death
Source: Front Neurosci. 2022 Mar 11;16:833630. doi: 10.3389/fnins.2022.833630 (PMC8963114; doi:10.3389/fnins.2022.833630)

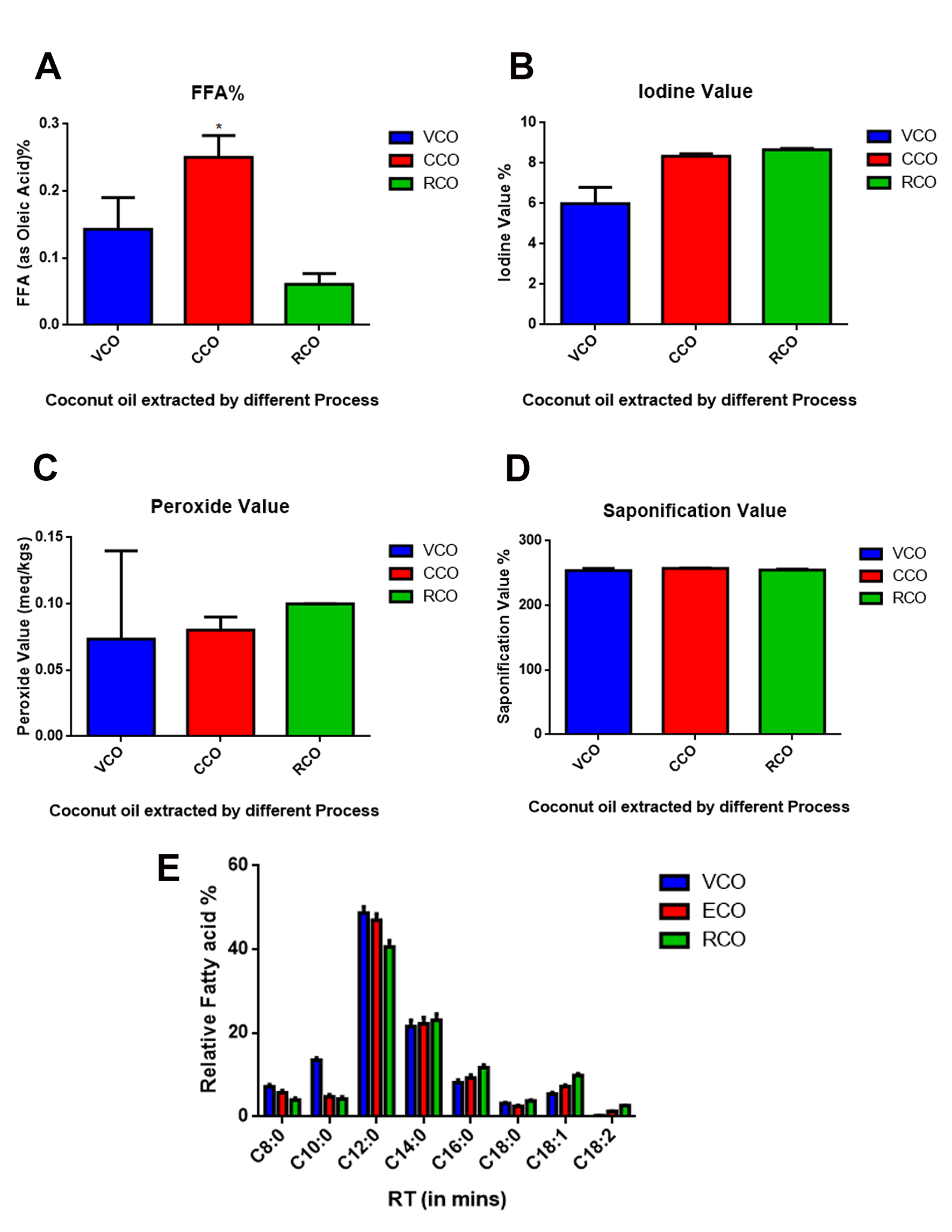

Supplement: Supplementary Figure 1 — Chemical parameters of VCO, ECO, and RCO. (A) Free Fatty Acid (B) Iodine Value (C) Peroxide Value (D) Saponification Value (E) Percentage Fatty acid composition determined using GC-MS relative to total fatty acid in VCO, ECO, and RCO. Data represent mean ± SD. [file Image_1.TIF]

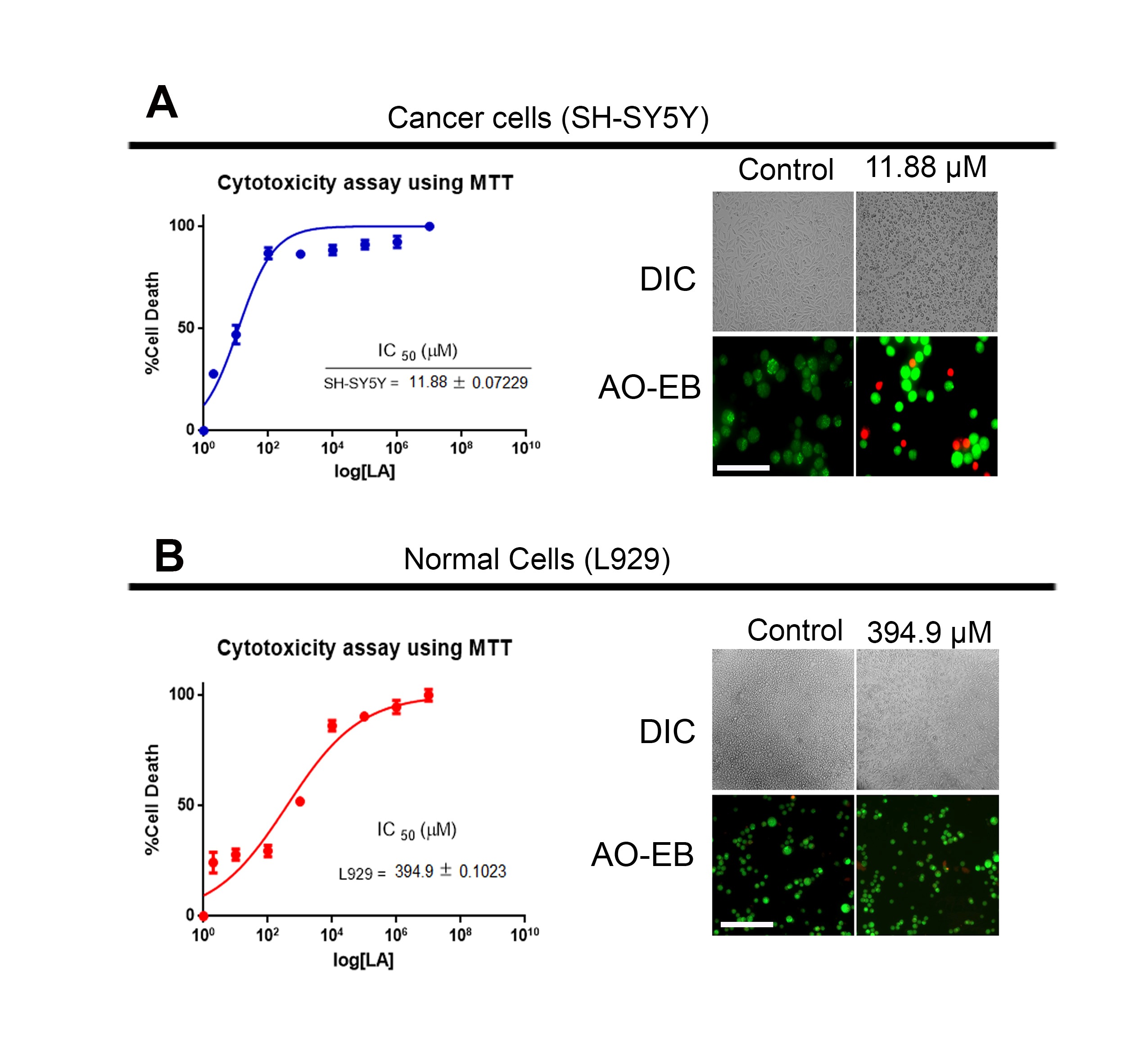

Supplement: Supplementary Figure 2 — Assessment of cytotoxicity of LA in cancer (SH-SY5Y) and normal (L929) cells. (A) Cytotoxicity of LA in cancer (SH-SY5Y) cells by MTT assay. (B) and photomicrographs showing LA treated SH-SY5Y cells, panel 1—DIC, panel 2–AO-EB staining (scale bar, 50 μm), (C) Cytotoxicity of LA in normal (L929) cells by MTT assay (D) photomicrographs showing LA treated L929 cells, panel 1—DIC, panel 2–AO-EB staining (scale bar, 100 μm). Data represent mean ± SD. [file Image_2.JPEG]

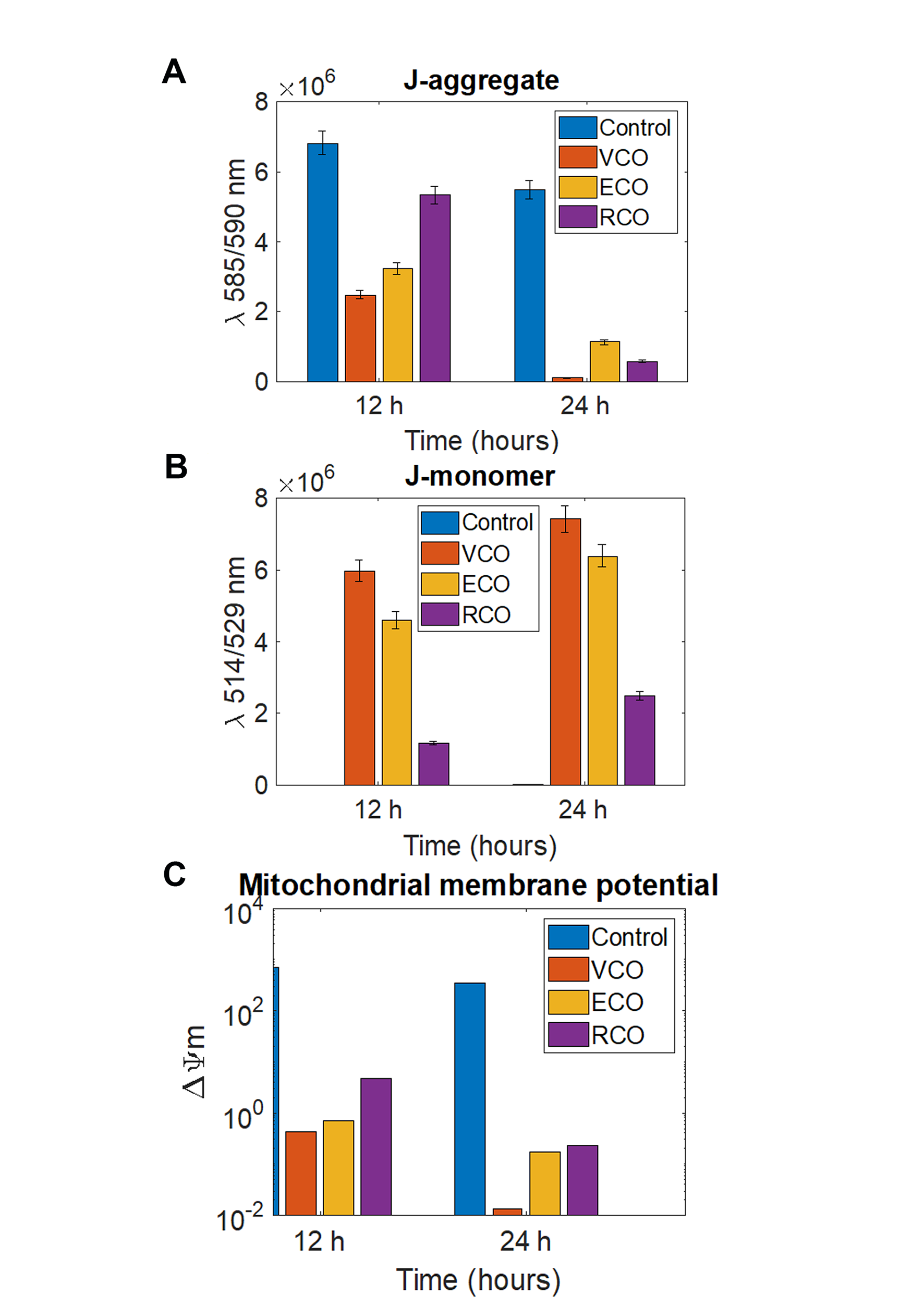

Supplement: Supplementary Figure 3 — JC-1 analysis of mitochondrial membrane potential in SH-SY5Y cells determined after 12 and 24h. (A) J-aggregate, normalized fluorescence intensity measured at λ590. (B) J-aggregate, normalized fluorescence intensity measured at λ520. (C) Mitochondrial trans-membrane potential (Δψm) calculated by a decrease in the λ590/520 ratio. Data represent mean ± SE. [file Image_3.TIF]
