# Supplementary material for: Dual Roles of Coconut Oil and Its Major Component Lauric Acid on Redox Nexus: Focus on Cytoprotection and Cancer Cell Death
Source: Front Neurosci. 2022 Mar 11;16:833630. doi: 10.3389/fnins.2022.833630 (PMC8963114; doi:10.3389/fnins.2022.833630)
Supplement: Supplementary file 4 [file Table_1.DOCX]

Supplementary Table 1. Primers for Real-Time PCR

| 1 | GCLC | Forward- | GATTGAAGGGACACCTGGC |
| --- | --- | --- | --- |
|  |  | Reverse- | TGTGCTCTGGCAGTGTGAAT |
| 2 | Ho-1 | Forward- | GAGCGAAACAAGCAGAACCC |
|  |  | Reverse- | ACCTCGTGGAGACGCTTTAC |
| 3 | Nqo1 | Forward- | CGAAGCATTTCAGGGTCGTC |
|  |  | Reverse- | AGATTCGACCACCTCCCATC |
| 4 | iNOS | Forward- | TGTGACACACAGCGCTACAACA |
|  |  | Reverse- | GAAACTATGGAGCACAGCCACAT |
| 5 | IL-6 | Forward- | CAAGAAAGACAAAGCCAGAGTC |
|  |  | Reverse- | GAAATTGGGGTAGGAAGGAC |
| 6 | TNFα | Forward- | TCCCAGGTTCTCTTCAAGGGA |
|  |  | Reverse- | GGTGAGGAGCACGTAGTCGG |

Supplementary Table 2.Physical parameters of VCO, ECO, and RCO

| Parameter | Virgin Coconut Oil (VCO) | Crude Coconut Oil (ECO) | Refined Coconut Oil (RCO) |
| --- | --- | --- | --- |
| Moisture and volatile matter (%) | 0.0838 ± 0.257 | 0.0872 ± 0.0048 | 0.0432 ± 0.0056 |
| Density (g/ml) | 0.906 | 0.906 | 0.906 |
| Refractive Index at 40°C | 1.4478 ± 0.0005 | 1.4480 ± 0.0013 | 1.4486 ± 0.0001 |
| Color (Y + 5R) using 1 inch cell | 0.13 ± 0.06 | 2.70 ± 0.26 | 0.67 ± 0.15 |
